# A three perspective study of the sense of home of nursing home residents: the views of residents, care professionals and relatives

**DOI:** 10.1186/s12877-016-0344-9

**Published:** 2016-10-03

**Authors:** J. van Hoof, H. Verbeek, B. M. Janssen, A. Eijkelenboom, S. L. Molony, E. Felix, K. A. Nieboer, E. L. M. Zwerts-Verhelst, J. J. W. M. Sijstermans, E. J. M. Wouters

**Affiliations:** 1 Fontys University of Applied Sciences, Fontys EGT - Centre for Healthcare and Technology, Dominee Theodor Fliednerstraat 2, 5631 BN Eindhoven, The Netherlands; 2 Fontys University of Applied Sciences, Institute of Allied Health Professions, Dominee Theodor Fliednerstraat 2, 5631 BN Eindhoven, The Netherlands; 3 Maastricht University, CAPHRI School for Public Health and Primary Care, Department of Health Services Research, Duboisdomein 30, 6229 GT Maastricht, The Netherlands; 4 Fontys University of Applied Sciences, Fontys School of People and Health Studies, Dominee Theodor Fliednerstraat 2, 5631 BN Eindhoven, The Netherlands; 5 EGM architecten, Wilgenbos 20, 3311 JX Dordrecht, The Netherlands; 6 Quinnipiac University School of Nursing, North Haven Campus, Office MNH 470P, 275 Mount Carmel Avenue, Hamden, CT 06518-1908 USA; 7 Fontys University of Applied Sciences, Institute of Human Resource Management and Psychology, Emmasingel 28, 5611 AZ Eindhoven, The Netherlands

**Keywords:** Sense of home, Older adults, Residents, Alzheimer, Caregiver, At-homeness, Homelike, Photography, Photovoice

## Abstract

**Background:**

The sense of home of nursing home residents is a multifactorial phenomenon which is important for the quality of living. This purpose of this study is to investigate the factors influencing the sense of home of older adults residing in the nursing home from the perspective of residents, relatives and care professionals.

**Methods:**

A total of 78 participants (*n* = 24 residents, *n* = 18 relatives and *n* = 26 care professionals) from 4 nursing homes in the Netherlands engaged in a qualitative study, in which photography was as a supportive tool for subsequent interviews and focus groups. The data were analyzed based on open ended coding, axial coding and selective coding.

**Results:**

The sense of home of nursing home residents is influenced by a number of jointly identified factors, including the building and interior design; eating and drinking; autonomy and control; involvement of relatives; engagement with others and activities; quality of care are shared themes. Residents and relatives stressed the importance of having a connection with nature and the outdoors, as well as coping strategies. Relatives and care professionals emphasized the role the organization of facilitation of care played, as well as making residents feel like they still matter.

**Conclusions:**

The sense of home of nursing home residents is influenced by a multitude of factors related to the psychology of the residents, and the social and built environmental contexts. A holistic understanding of which factors influence the sense of home of residents can lead to strategies to optimize this sense of home. This study also indicated that the nursing home has a dual nature as a place of residence and a place where people are supported through numerous care strategies.

## Background

Nursing homes provide an alternative place of residence where 24 h care and assistance is offered by professionals when people can no longer reside in their own home environment due to increasing need for assistance with activities of daily living, complex health care needs and vulnerability [1, 2]. Admission to a nursing home is a major life-event, as most individuals do not wish to leave the home they have been living in for a long time in order to move to a nursing home [3]. Nursing homes have a dual nature: as a care institution and as a place to live. Therefore, many health care organizations try to provide living arrangements that focus on ‘the good life’ and the creation of an environment that is like a home to its residents, instead of a health care facility in which they also reside [4]. Delivering both good (clinical) care and a homelike environment is challenging. Nevertheless, “*there seem to be good reasons to assert that living in an institution and being “at home” is not a contradiction in terms*” ([5], p. 221).

One of the challenges in modern day nursing home care is to create a sense of home for the residents. The sense of home is a multifactorial phenomenon which is highly influenced by both social and personal characteristics, as well as the built environment and the architecture of the facility. A sense of home is related to personal experiences and emotions, and does not come into existence over night, but is gradually developed by the person for whom independence, security and the source of own identity, choice and controls, as well as memories are essential [6–14].

Rijnaard et al. [10] systematically reviewed the factors influencing the sense of home of older adults residing in the nursing home. Their review showed that the sense of home of nursing home residents is influenced by as much as 15 factors, which are divided into three themes. The first theme consists of psychological factors, including the sense of acknowledgement, preservation of one’s habits and values, autonomy & control, and coping. The second theme consists of social factors, which include interaction and relationship with staff, residents, family & friends, and pets, as well as specific activities. The third theme is the built environment, which includes the private space and the (quasi-) public space, personal belongings, technology, the look and feel, and the outdoors and location of the nursing home. These three themes are a representation of the dimensions in which nursing home residents experience or develop a sense of home. Molony [7] also included the centrality of home as a both a refuge and link to personal meaning, and described the dynamic nature of home as a temporal process of person-environment integration signified by an openness to new possibilities in the current environment and situation.

Insights into the differences in perspectives on the concept of home are sparse. In practice, various stakeholders are involved in the creation of a sense of home, such as the residents themselves, their professional caregivers working in the nursing home, and relatives of the residents. To the latter two groups, the nursing home environment is either a place to work, or a place that should be inviting to visit and assist their loved-ones. Differences in the perspectives and insights of these three groups, may lead to suboptimal conditions for any of these groups, as preferences or needs of one group prevail over those of the others [15, 16]. If creating a sense of home is recognized as the central goal, the needs and preferences of the residents themselves must be prioritized.

This study explores which factors in the psychological, social and built environment are related to the sense of home of the residents. The goal of this study is to gain insight into the experiences and views of actual residents, their relatives and care professionals, in order to understand their needs in relation to the design of nursing homes and to promote a social context that facilitates person-environment integration. In order to carry out the multi-perspective study, three separate research questions are defined.

For residents: *“Which aspects create an optimal sense of home in the nursing home for you?”*

For care professionals: *“Which aspects contribute to a sense of home of nursing home residents, and how can care professionals help create these things for the residents, and, at the same time, have an optimal place to work?”*

For relatives: *“Which aspects contribute to a sense of home of your loved-one, and consequently, what makes it a place you like to visit?”*

The focus of the three research questions is on personal, social, and physical aspects, in existing nursing homes, and on things one can do, experience or organize with a limited amount of (financial) means.

## Methods

A qualitative methodology was chosen for this study, incorporating photography, in depth interviews with nursing home residents, and focus group sessions with relatives and professional caregivers. The Critical Appraisal Skills Programme’s checklist for qualitative research [17] was used as a guide for this study. In the following sections we describe (1) the settings, participants and ethical considerations, (2) photography approach, (3) interviews, and (4) the data analysis. This study builds on the foundations laid in the study by van Hoof et al. [14].

### Settings and participants

**Fig. 1:**
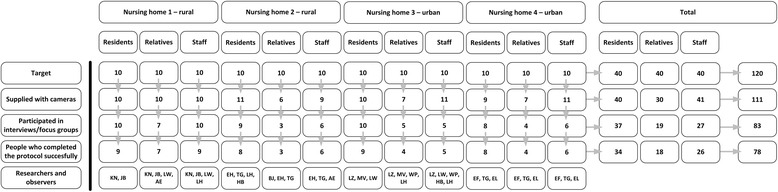
Number of participants per nursing home

In October and November 2015, a field study was conducted in four facilities of four nursing home organizations in towns in the south of the Netherlands. Two of these nursing homes were urban and two were rural nursing homes, in which residents have a private room. The resident populations were mixed, i.e., resident had either a psychogeriatric, physical or combined diagnosis for admission. The study aimed to include both residents with physical limitations and psychogeriatric health problems, as well as informal caregivers (relatives) and professional caregivers, in order to gain a wide set of perspectives. The inclusion criteria for the research were as follows; residents had to be at least 55 years of age, had to reside in a nursing home for at least six months, had to be able to communicate in Dutch, had to be able to take pictures with a photo camera independently or with the help of caregivers or relatives, had to be able to make a selection of important photos independently, and had to be able to have an interview of at least 30 min. Informal caregivers had to have a sustainable relationship with a nursing home resident (in this study: family members). Professional caregivers, in this study limited to nurses and nursing aides, were invited to join. For every of the four nursing homes, the research team aimed to recruit 10 residents, 10 relatives, and 10 care professionals, totaling 40 participants for each group (Fig. 1). In the end, a total of 78 participants successfully completed the study protocol (*n* = 34 residents, 18 relatives and 26 staff). The main reason for attrition among relatives and staff was the lack of time to participate both in the field study and in the subsequent joint meetings.

### Photography

A visual research method was chosen for this study [14, 18, 19], in which people are interviewed based on photographs they have taken themselves with cameras supplied to them by the research team. Photography is a method that has been used in research for decades, and which allows participants to create a record of an event, capture a complex phenomenon or to tell a story through images [20–22]. As taking photos does not rely on language alone, it can be used with vulnerable or frail populations who might not normally be included in research [20–22]. Images provide a lasting record of an event, or in the case of this study an architectural or social scene, required to study the relationship of people and their living environments. A photo only serves as a conduit to enhanced, thoughtful, and deliberative narrative, instead of as a replacement of words. Photography allows complex environmental, health and social issues to be captured and then shared with other people [19, 20]. As this study tries to capture the real-life experiences of nursing home residents, the photo-production method was applied [18]. In line with Annemans et al. [19], this study sought to obtain a good understanding of what has been made visible on the photos, in addition to why and how. The photo production method enables researchers to experience a phenomenon from another point of view, in other words, the nursing home as a living environment.

### Joint procedure at the start

Every field study started out by having an introduction presentation in the four nursing homes, in which information about the research was provided to potential participants. At the kick-off presentation, prospective participants of all groups received an information letter from a member of the research team. Informed consent was obtained from the participants (for residents, in conjunction with their initial family caregivers) by signing the given consent forms. Nursing home residents, their relatives and care professionals were invited to make pictures of what gives the residents a feeling of home in the nursing home. This could be typical activities that provided a sense of home, or typical aspects of the built environments; in essence everything that visualized the experience of being at home in the nursing home. A manual including instructions how to take the photos supplied to all participants. Participants were asked not to take explicit pictures or photographs of people who did not want to be photographed, as photographs would be shared in focus group sessions within the own nursing home and become available to the researchers for analysis.

The research team chose to provide the participants with disposable cameras instead of digital cameras, as laid out by van Hoof et al. [14]. Disposable cameras are cheaper than digital cameras and when dropped, are not as easily damaged. In this study, participants were supplied with a camera for one week up to a week and a half, and were asked to take pictures of their living environment. The reel of the camera contained about 25 photos, which should be sufficient for taking the most important pictures, but participants were free in the number of pictures they took (taking fewer than 25 photographs was allowed). Support to residents could be provided to the participants for taking the photographs, for instance, by relatives, staff or interns working in the facility. After the time period, the cameras were collected by the research team, and sent off for development of the photos. Upon completion, the principal investigator returned to the participants. The photos were used as a basis for the interviews (residents) and focus group sessions (relatives and staff), as participants could use these photos as a foundation for what they wanted to discuss in the interview [23].

### Interviews with residents

Individual interviews with residents were conducted, during which residents talked about their photographs, and how they attributed social and personal meanings and values to these photographs. This individual approach was chosen because the research team wanted to maximize the input for every resident in a secure setting. The interviews had the character of a conversation and varied between 30 and 60 min each, depending on the richness of the conversation and the attention span of the participants. Each interview commenced with an introduction and the opening question included whether participants could describe their experiences concerning their living environment in general. Thereafter, the pictures were discussed in terms of the contents of the photographic material. Participants were asked to describe what the pictures meant to them in the context of the sense of home. The interviews were supplemented by items from a topic list, which was based on the work of Van Steenwinkel et al. [11] and van Dijck-Heinen et al. [4]. The participants were free to add other topics related to the concept of home, which were not included the topic list.

### Focus group sessions with relatives and care professionals

A total of eight joint focus group sessions were held in every nursing home with (1) relatives, and separately with (2) care professionals. This group approach with either relatives or care professionals was chosen because the research team wanted the participants to interact and discuss their findings. The focus group sessions lasted for about 90 min each. During the session, photos of the participants were viewed by each other, and discussed. Pictures and comments were thematically clustered by the participants. Based on the discussions, participants were invited to write down which positive aspects of the nursing home should be kept, and which aspects should be improved. These aspects were later discussed in a plenary session with the participants. Subsequently, a prioritization of positive and negative aspects was made by the participants, in order to have direct take-home messages for the participants.

### Data analysis

All interviews and focus groups were recorded with a voice recorder, and transcribed verbatim by the research team. The data were analyzed based on the six phases by Braun and Clarke [24]. First, all transcripts were read in their entirety. Then, the first set of codes was generated through open coding. The researchers, bearing the research questions in mind, systematically highlighted the relevant information (open coding) in the transcripts. Open coding concerns the process of unravelling all of the collected data into fragments or codes. Similar codes and quotes were clustered and labelled, and themes emerged from this process. Together, the research team organized the codes and clustered them into smaller thematic groups on a plenary research team meeting in December 2015 (axial coding). During this session, the codes from the four nursing homes were written down on post-it® notes that came in four separate colors, and were clustered on three sheets, representing the three groups of stakeholders. Themes were formed out of the codes during a plenary discussion sessions with EZ, KN, BJ, EF, JH, JB, MVe, and which were observed by MVo. These themes were reviewed, and then defined and named. A second plenary discussion with the research team was held in March 2016 in order to come to a final consensus on the themes and factors and selective coding was applied. In order to guarantee the anonymity and privacy of participants, names of people and institutions, appearing in quotes, were deleted from the written texts or were changed.

## Results

**Fig. 2:**
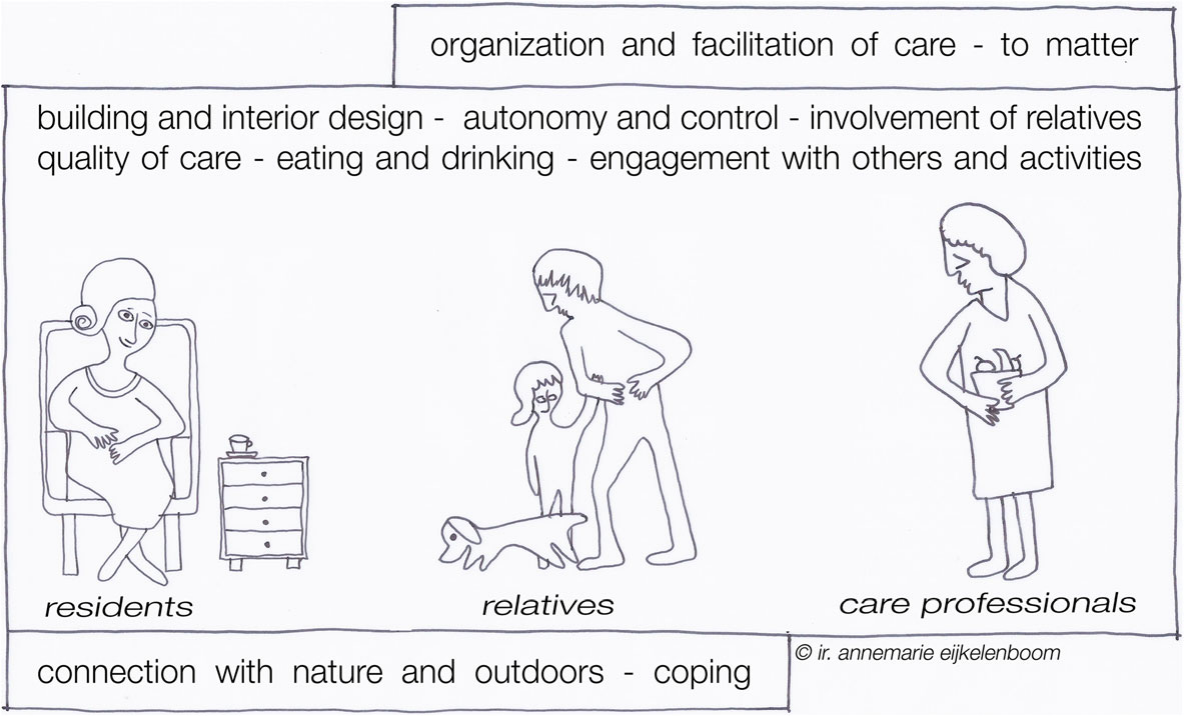
Graphical overview of themes for the three stakeholder groups

There are numerous factors that influence the sense of home. The analysis led to the identification of several themes, which overlap between the various groups of stakeholders, but which have subtle differences in their constitution (Table 1, Fig. 2). Most of the photos taken by the participants were used as auxiliary material during the interviews. The overall quality (colors, focus) of the photos was too low to be included in this section. The overall impression of the photographic material was the same throughout the groups, but the meaning given to the photos differed.

**Table 1:** Overview of themes per stakeholder group

Residents	Relatives	Care professionals
Building and interior design	Building and interior design	Building and interior design
Eating and drinking	Eating and drinking	Eating and drinking
Autonomy and control	Autonomy and control	Autonomy and control
Involvement of relatives	Involvement of relatives	Involvement of relatives
Engagement with others and activities	Engagement with others and activities	Engagement with others and activities
Quality of care	Quality of care	Quality of care
Connection with nature and outdoors	Connection with nature and outdoors	
Coping	Coping	
	Organization and facilitation of care	Organization and facilitation of care
	To matter	To matter

These themes are further elaborated in the following sections.

### Building and interior design

Both the interior and exterior design contribute to a sense of home of all three groups of stakeholders. Residents cherish being able to bring their own possessions and turn the room into a familiar place. The nursing home should have a practical layout which is supportive to the resident. More rugs, candles, flowers and colors are desired, or more practically, nice placemats on tables. Residents wish to be able to have visitors in their private room. Personal possessions that remind people of their past are appreciated, such as paintings, old chairs and even television sets. Some residents do not have a private shower and indicate that this hampers their sense of home. These residents also wish more closet space, and kitchen areas are not always needed in the private room as staff takes care of the cooking. Some residents wish for a larger room. Single person’s rooms are preferred, full of paintings and photographs that remind people of their past. Empty walls are considered unattractive, and some of the residents stated that the interior design of the communal areas is dull and frugal. Privacy in terms of the built environment is mentioned as well.

*“Yes, my room is a bit small. Not so large. But still, it is my own stuff. It is my own and it is safe from others.” [resident]*

*“I brought as many things from home as I possibly could.” [resident]*

In terms of the organization of care, in one nursing home people mentioned the fact that they are not allowed to put nails into the walls to hang pictures as a negative feature that hampers a sense of home. Residents want to stay in control of their own room and the way it looks.

*“It is important that my table is full of stuff, and that I am allowed to leave it full of stuff.” [resident]*

The relatives in this study stressed that having a spacious room is a facilitator to the development of a sense of home. The interior design and furniture should be familiar and preferably brought from the old home.

*“Now that the room is all decorated, she has a peace of mind. And she is very content.” [relative]*

In terms of a familiar interior design, relatives speak of chairs, special crockery, and having a private chair in a preferred spot. There should be sufficient closet space, display space, and storage for personal items. The building should be designed in such a way that care professionals cannot lock themselves up, away from the residents, and that they can be approached by others.

*“The office of the care staff is always locked. There should not even be a door! Always shut! It is all covered up. You cannot see them sit in there.” [relative]*

Certain facilities, including Wi-Fi, should be available throughout the entire nursing home building. Relatives are aware that there is a need for privacy, but stated that this may be in conflict with the need to safety and security. Other relatives stated that residents should have their own spot in the communal space, and that there should be sufficient space to move and walk around.

*“Having your own key is essential. I now have the key to my mother’s room, we always had a key.” [relative]*

Some of the design could be nostalgic, with a regional or geographical touch, and based on the time-frame of the residents. In one of the nursing homes, residents were explicitly invited to decorate their own rooms, as well as the communal living areas.

*“One of the residents said: ‘Finally, I have my old belongings back again.’ It was actually stuff from a second hand shop.” [staff]*

*“And this hospital corridor? It absolutely does not allude to home. I think this corridor represents what people do not want. It symbolizes a sense of institution.” [staff]*

When decorating a space, aspects of accessibility should be considered, also in terms of the layout of a space and the presence of thresholds and other architectural features that impede or support accessibility. Human factors and ergonomics are integral parts of the built environment, according to the care professionals. A circuit for walking was mentioned by the care professionals as an important architectural feature for residents to feel at home. In this circuit, there should be attention for design and decorating, as well as having various objects and cues for orientation. Having the right lighting is important too.

*“Some group rooms are too small to accommodate the residents. And many relatives take their loved-ones to their private rooms.” [staff]*

*“I don’t understand that you put all these obstacles in the corridor. Lately, the corridor has been empty, and I thought that was a positive thing.” [staff]*

### Eating and drinking

Residents can really enjoy food and drinks. Being able to cook or prepare meals oneself is valued by some, but at the same time many residents have never cooked themselves and do not venture out in the kitchen. Some residents explicitly stated that they like that fact that others cook for them. Instead of helping to prepare dinner, they ‘helped’ eating whatever was cooked for them.

Drinking coffee together is a moment cherished by many. Sitting around a table together reminded many people of the old days when they did this with their large families.

Mealtimes, food and drinks are recognized by relatives as an integral part of the sense of home, and this group of stakeholder stressed the importance of food and drinks the most. A full fridge represents a family atmosphere, a sense of affluence, and people have the notion that it is okay to take something from the refrigerator.

The smell of food contributes to a home-like atmosphere. Apple pies, deep-fried snacks, fries, barbecued foods, are mentioned by relatives. Some also think it is acceptable that people get alcoholic drinks at 11:00 in the morning. Residents should be able to eat normal means and have some choice of what they want to eat. Tables should be set according to personal preferences.

*“My husband hates mashed potatoes. He likes boiled or baked potatoes, but no mash. He simply can’t swallow it. And still he gets the mash.” [relative]*

Some of the mealtime portions are not fitting. At home, people had more of luxury food items than that they are allowed to have in the nursing home.

*“If you live at home and you like this raisin bread, you just eat the whole loaf. Mum asked how much she was permitted to have. Staff told her that all she could have was one slice.” [relative]*

Staff regarded food and drinks integral part of the sense of home. Eating is both functional and a social activity.

*“Sitting around a table together. And having conversations together. It is something you do at home. The larger the seating arrangement, the less people talk” [staff]*

Dining is more than just eating; it is also a moment of peace, and a part of the daily routine and autonomy. Ambiance is a part of the dining experience. In small living rooms, there is no need for a meal cart. A refrigerator is mentioned by a number of participants, also as a sign of hospitality. Being able to choose what you want to eat and when, partly symbolizes the sense of home, such as opening a pack of crisps or make a cup of warm chocolate. Furthermore, residents can help in preparing food and setting plates.

*“What I find important at home, is a refrigerator full of food and drinks that I like.” [staff]*

*“Just being able to pick fruit from a table. It is there for people to take. And they do! And you shouldn’t feel bothered if you find an apple that is missing a bite…” [staff]*

### Autonomy and control

Autonomy is mentioned by the residents as an important factor in their sense of home. People should ideally be equals, and should have a freedom in the choices they make, and have some degree of influence in aspects that impact their daily lives. However, residents experienced low levels of autonomy, which impeded their control and sense of being at home. Being able to assist in household activities is appreciated, but is not appealing to all residents. Many residents find it an attractive idea that many tasks are being taken care of.

*“Things are always done for you before you can decide yourself. When you are at home, you make decisions yourself. Now, when something needs to be done, you need to talk it through and then there is a meeting”. [resident]*

When speaking to the relatives, they stated that there should be room for the values and standards of residents. Unnecessary rules should be abolished. Residents should be able to behave like they did at home. They should be free to read a newspaper or watch television. At home, residents were also in charge of their daily lives. People should be allowed to smoke a cigarette. A resident’s autonomy should be respected as much as possible, with a minimum of interference, not being forced (to eat, drink or wear certain outfits), and to make your own decisions in life, despite obvious financial constraints.

*“No, there is no such thing as self-direction here. After 5 o’clock things shut down in this building. The door is simply being locked. You don’t lock your own living room at 5, do you?” [relative]*

*“Even though my mother wants a specific type of incontinence material, she gets another type she does not want. As the other type would be too expensive, and is available for nighttime only. This is not beneficial to feeling home.” [relative]*

The autonomy of residents is mentioned by the professional caregivers as important for the development of a sense of home. The degree in which residents have control over their own lives may be in conflict with the rules set out by the organization, the resident’s independence and the limited understanding of one’s physical or mental condition. The care professional work with the best of intentions and try their best to care for the residents, and make them feel at home. At the same time, they have a care schedule full of tasks to complete.

*“There is difference in existing and new clients. In our ward, the recent arrivals want to go to bed late.” [staff]*

*“Yes, we all say that people are free to do this and that. These are nice phrases and intentions, but it cannot be realized in practice” [staff]*

### Involvement of relatives

Having family around you, whether it is through having photographs in the private room, by making telephone calls or by having relatives visiting, is important to a vast majority of residents to experience a sense of home. Real visits by relatives cannot be replaced by photos or telephone calls. Being visited by grandchildren is appreciated, but at the same time there should not be too many visitors at the same time. Being visited by a spouse and doing things together, such as making outings, is valued. Many children take care of small tasks as well. Being visited by relatives seems to be more important than being assisted by them.

*“This place needs to be as attractive as possible for the children”. [resident]*

*“If I want see my daughter, all the way in New Zealand, then I can use a computer. I can use Skype”. [resident]*

Relatives wished to participate in the lives of residents. Being extended a warm welcome or being greeted by staff is essential for relatives. So is having a high quality relationship with staff. If relatives felt welcome, they were inclined to visit more often. The fact that in some nursing homes, they are allowed to take coffee themselves and that they feel welcome, are incentives for visiting. Some relatives mentioned the distance between relatives and staff, like there is an ‘us against them’ situation. Relatives feel that they need to step up for their relatives and give them a voice. Relatives want recognition as an expert, too. At the same time, relatives expressed that they have insufficient knowledge of treatment of their loved-ones by the staff. Relatives worry about the negative image of the nursing home in the community and hope to contribute to a more positive image themselves.

*“In the early days when we played cards with my uncle, we brought our own coffee. The staff asked us what we were doing. We answered by saying that we were not allowed to take coffee ourselves. And then the staff responded by saying that that was about to change right away! Ever since, we were allowed to make coffee ourselves.” [relative]*

*“Having to fight for your loved-one has nothing to do with a sense of home. It is a pile of frustration. Which keeps on piling up.” [relative]*

Staff said that it is important to engage in a dialogue with relatives, and to inform relatives as much as possible about the situation in the nursing home. Hospitality should be offered to relatives and other visitors. Relatives should not experience any barriers when wanting to approach a member of staff. The relationship between staff and relatives is not always optimal, and offers room for improvement.

*“I wish we had a positive collaboration with the relatives (laughter)… I would like that so much.” [staff]*

*“The moment you enter the room, and you see the family sit together with mother, and they bring something nice to eat, and the daughter is making a cup of tea. Yes I love seeing that [as a care professional].” [staff]*

### Engagement with others and activities

Residents wish to engage with others in a social manner and social contacts are an essential part of life. It is appreciated when people can walk in and out of the ward in order not to feel lonely. Residents wish to be part of a group, although being together with others also means giving up some degree of privacy. Despite living in a group, residents can still experience feelings of loneliness or being excluded from a group. The quality of these social contacts is rather an essential element to the residents. Engagement with other residents takes place when having conversations, when eating together, although it happens that other residents are not interesting enough to interact with.

*“In the living room, people often sing songs. I sang along aloud and danced a lot”. [resident]*

*“A sense of home is being together”. [resident]*

People wish to participate in activities to engage in a meaningful daily life. Preferably, these activities are something residents did in their earlier years. The residents also wish to be able to plan themselves how they wish to spend their days. Some explicitly stated that they have worked all of their lives and that they like that they are being taken care for. Habits and routines are mentioned as important aspects of daily activities. Organized leisure activities were mentioned to give distraction, although residents wondered if these were useful and meaningful. Activities people conducted themselves were reading, listening to music and playing puzzles, as well as drinking coffee and cycling. Some also mention assistive technologies which are needed to continue doing enjoyable activities.

*“In the morning, I spend my time cleaning.” [resident]*

According to the relatives, having social contacts is considered to be essential for having a sense of home. Doing all types of homelike and household activities are opportunities to do things together with others. Relatives mention the presence of inviting meeting points as an opportunity for engagement, for instance, the fire place or the kitchen table. Residents should be able to receive visitors if they wish to. The stimulation of senses in the communal rooms, for instance, through music, odors of cooking, making paintings and touching each other gently, is mentioned by relatives. It is important that there is sufficient attention for each other and that people have a sense of belonging. Negative aspects are unwanted contacts, of having to engage with people you did not choose yourselves. In general, relatives stress that there is insufficient engagement with co-residents, and that most engagement is with relatives, pets and acquaintances.

*“When no one comes to visit, it can be the most homely place but it is no good.” [relative]*

According to staff, residents should be stimulated to participate in doing activities, which can be done in a group or as an individual. The care professionals also stated that the nursing home should be part of a larger community, and the outdoor world should be an integral part of life in a nursing home.

*“We should bring the neighborhood indoors!” [staff]*

According to staff, daily rituals are important for a sense of home. Engaging in familiar household activities indoors and outdoors is a form of introducing familiarity, which, in turn, contributes to sense of home. Residents should be able (or stimulated) to recognize things, including the layout of the ward, things from their past, activities, and personal items. Providing residents with structure, and a small-scale environment helps to create familiarity. Being familiar with the professional caregivers is mentioned by this group as being important: residents should know who looks after and cares for them.

*“I often think of them as a group of people. All of them are individuals, and came here with a totally different background, and still they live in a sort of family situation. And when someone passes away, there needs to be another place for someone else.” [staff]*

*“A sense of home. Familiar faces. All very important. Well, this is something we hear often from residents at the moment. ‘Another stranger again!’ ” [staff]*

### Quality of care

Care should be individualized as much as possible, according to the residents, in order to feel at home. Residents experience a continuous tension between trust and mistrust towards care professionals, though the help of care professionals is highly valued. There should be space for culturally different emotional expressions. The treatment of residents should be more mature. The experience of safety and security is an important feature of care professionals according to the residents. Volunteers are important, as they make soup or read a newspaper. The characteristics of care professionals are mentioned often, for instance, being open to talk about problems and being a good aide. Resident talked around the issues of conflict and critique, because of their dependence.

*“Of course, I can say ‘no’ [to the care professionals], but when you need their help at a later moment, then they will turn down on you, too.” [resident]*

*“The mood the staff is in is important.” [resident]*

Despite some of the criticism of residents, overall, a professional approach to care is valued, as it is also a source of security and peace to the relatives. Relatives indicated that there should be sufficient trust in the caregivers that they execute –at least- the essential elements of basic care. Relatives wish that they have a say in how their loved-ones are cared for, and wish to have a voice in the treatment and approach to care. The quality of care also involves the approachability of staff. Some relatives understand the negative behavior of residents and have empathy for their loved-ones given the situation. According to the relatives, there are certain characteristics of qualified staff, namely, being motivated, having an eye for the emotions of relatives, being able to create a positive atmosphere, being a good colleague to other staff members, considering the values of individual residents, and having a sufficient degree of education. Moreover, a good care professional is able to communicate with relatives and enter a dialogue. There should be room for the own initiatives of relatives. The overall atmosphere of a nursing home is very important, people wish to be part of a community, which feels safe and secure, and which offers opportunities to do typical things you do at home. There needs to be a sense of trust that their loved-ones are in good hands.

*“As long my mother is taken care of well, I feel comfortable too.” [relative]*

*“Staff are the most important! They take care of the atmosphere in the nursing home and that is so important.” [relative]*

When relating to the quality of care, care professionals stated that their empathic abilities and ‘having a heart’ were of great importance to the development of a sense of home. The factors of the quality of care could be best described as the core of the care professional: head, heart and hands. Staff stated that they need to get to know the residents and their backgrounds. They can contribute actively to the atmosphere in the groups of residents, by being near, by showing signs of intimacy, or by the provision of undivided attention (not having to share time with others), comfort and a sense of security to the residents.

*“It is about being able to put a smile on the face of the resident. It is the small things that matter most.” [staff]*

*“You can have a lot of colleagues with many qualities, but if they don’t have a heart for my residents, then I don’t want them to be around me too.” [staff]*

### Connection with nature and outdoors

This theme emerged only from the data from residents and relatives. Many residents wished to be able to go outdoors, or appreciate the view through the windows, and be connected to nature. A view of green spaces, trees and gardens is mentioned by residents from the rural nursing homes. People wish they could walk around outdoors. Animals in the garden are appreciated. Some residents used to live in a farmhouse and animals were an integral part of farm life. In the rural nursing homes, residents wished they had more shops around. A view, whether connected to nature or otherwise, is generally appreciated.

*“Yes, I love being outside”. [resident]*

*“I have a good view of the road and the round-about. So from that chair I can see everything, and I like that”. [resident]*

Having access to a garden as in the old days is mentioned as being important in rural nursing homes, according to the relatives. A nice view, with animals, plants and nature is mentioned by relatives in one of the urban nursing homes. Liveliness, animals (bird cages, chickens) are important contributors to a sense of home. In case of one of the nursing homes, the surroundings are a forested area which is ideal for walking. Keeping a vegetable garden is appreciated, especially when what is produced is eaten by the residents.

*“This is the new greenhouse of the nursing home. She always had one herself at home, as well as a vegetable garden. Until a year ago she had chickens as well. About three. And she always had fresh eggs. To her this is a homelike thing.” [relative]*

*“I was not allowed to bring my dog. The ‘non-sense’ of home.” [relative]*

### Coping

This theme emerged only from the data from residents and relatives. Many of the residents expressed that they have trouble getting used to living in a nursing home but that they have to cope. The old home will forever feel like the last true home to many. Some try hard to make the nursing home feel like home, for instance, by decorating the rooms themselves. Apart from losing a home, moving also means losing a spouse who was always nearby. Some spouses have died in the meantime as well. Being accepted for co-residents makes up for some of the losses. People should be able to feel meaningful, which is part of the coping strategy and the development of a sense of home. There are many ways to cope with the new situation, for instance by accepting the new living situation, its social context, being dependent of others and because of physical limitations. Instead of coping, some of the statements sound more like resignation or acquiescence.

During the interviews, many resident showed sign of strong emotions, which they tried to hide. Some people like to return home during the weekends.

*“Well, it is nice here, but I will never get used. I’d rather return to home, but it increasingly getting difficult. You just know you no longer can do it.” [resident]*

Relatives saw that their loved-ones are trying to cope with the fact they now live in a nursing home. Over time, they see that their loved-ones are trying to regain some of their old routines, such as cleaning and dusting. Some regain some sense of home, as they experience a sense of security to engage in small tasks. Some people cannot cope with the new living conditions, will not accept the new living situation and wish to return home.

*“She always did the [cleaning] at home, but not for some time when she came to live here. But now, she speaks of this place being her home. She no longer needs to leave. And when she came to accept her conditions, she regained some of her old tasks.” [relative]*

### Organization and facilitation of care

This theme emerged only from the data from relatives and staff. Relatives wished to see familiar faces and people in the wards. Relatives like to engage with a fixed group of professionals, in which there is a limited (natural) personnel turnover. Care staff should be easy to approach, also in terms of the building itself. Being locked up inside a nursing station is not appreciated. Relatives wished that it was possible to keep spouses together in the nursing home, and that this was facilitated by the organization. Relatives mentioned that writing reports and using computers in the presence of residents turned the home into an office, even though the caregiver was present instead of being at work in a separate room. A nursing home should also be a community that feels safe and secure, and which offers opportunities to do typical things you do at home. Relatives wished for private rooms and closets that could be locked, as belongings got lost.

*“The office of the care staff is always locked. There should not even be a door! Always shut! It is all covered up. You cannot see them sit in there.” [relative]*

Staff indicated that they need time, hands, financing and interns to provide adequate care to their residents. Only if these preconditions are met, can they feel being able to provide a sense of home to the residents. Technology is seen as a support tool for care, communication and activities, but education and training is needed. Other requirements are having a sufficient amount of parking space for the car (no need to be stressed in the morning when having to look for a free parking space), having a lack of managerial support, and having sufficient storage space for work-related items. Some members of staff take the freedom to buy items to improve the interior design of the ward.

*“Everyone who needs something from you, clings to you. And so that makes you react differently towards other people. You are becoming curt with someone, as there are a hundred thousand other things waiting for you because it is you and four other flex workers.” [staff]*

### To matter

This theme emerged only from the data from relatives and staff. According to the relatives, residents should be heard and should still ‘count’ or ‘matter’. There should be sufficient attention for the consequences of loneliness. Residents should be able to continue living their old lives with their old habits. Residents are entitled to live a life, despite a possible dementia diagnosis. Every person has certain qualities that can still be appreciated by the nursing home organization. Residents have the right to be heard, observed and recognized and acknowledged. Being dressed correctly and wearing make-up are integral aspects of this theme, although the terminology of dignity was not mentioned directly by the participants.

*“Well, this man has his private room, but he sits in the living room all day long and he says nothing and no one says anything to him because he gets completely ignored because he cannot speak.” [relative]*

The well-being of residents is mentioned in all three focus groups. All residents have the right to exist, and should not feel as a person that is no longer part of society. Care professionals should underscore/ enhance to the residents their sense of meaningfulness in order to provide them with a sense of home. This can be achieved by giving responsibilities to residents is a part of the set of tasks for the care professional, for instance, taking care of plants or being able to decorate a room according to one’s own preferences.

*“The people who are with us are taken out of society. They are placed within the walls of an institution, and they, if it were, I’m sorry to say, stop existing. […] The only thing they still have are the nurses who are there for them every day. […] They should have a connection to society, and have the feeling that they still exist”. [staff]*

## Discussion

This study found that residents, their relatives and professional caregivers all found 6 aspects important in contributing to being at home in a nursing home. These include a familiar interior design and building, eating and drinking in ambience, having autonomy and control over daily life, involvement of relatives, being engaged with others and in activities and having a good quality of care. In addition, for residents and their relatives it was important that they experienced a connection with nature and outdoors and that they learned to cope with the new situation. Professional caregivers and relatives stated that the organization of care needs to facilitate the sense of home and that it is important for them to make residents’ matter.

### Reflections on the results

The factors identified in this study seem to have a relationship with the experienced sense of home in this study seem consistent with the findings of earlier studies, for instance, the systematic review by Rijnaard et al. [10] and the study by Sixsmith [25]. This field study showed that the themes identified by the study by Rijnaard et al. [10] are also valid for the Dutch context. For instance, dining at the table like residents were used to, participating in household tasks, having access to a garden and familiar interior design. The relatives wish to be involved by the care professionals and to be informed about what is going on in the facility. Having a mutual conversation about needs and wishes can help the relatives to feel more free and more at home Intervention programs to increase family and staff have been developed, such as the Partner in Caregiving program [26]. The Partner In Caregiving program facilitates positive relationships between family caregivers and professionals through intensive training on effective communication, empathy development and conflict resolution. Positive effects such as improved attitudes and reduced conflict with family have been reported [26, 27]. Furthermore, in order to provide person-centered care, assessing and honoring residents’ preferences is a fundamental step, especially in relation to interpersonal relationships, coping strategies, personal care and healthcare discussions [28]. Evidence based tools are being developed to assist this process, such as the Preferences for Everyday Living Inventory [29], in which staff, family and residents reflect on personal preferences in the care process. Moreover, communication strategies should direct attention toward the person living in the nursing home and what they are capable of, as opposed to focusing on the disease and the care tasks required [30].

The atmosphere within a nursing home environment should be good, and this is facilitated by giving the residents as much free space as possible, allowing them to make their own choices. Caregivers need to take sufficient time to get to know the residents, and should be empathic at the same time. The creation of a homelike atmosphere is important, without making the environment look too cluttered. Family involvement in order to support the resident is an aspect mentioned by the care professionals. Tuning with relatives and offering hospitality are mentioned, as it may help relatives feel at home as well. The most prominent difference is the stress the three groups of participants in the current study put on food and drinks, and the specific attention for the organization of care and the impact an adequate facilitation of staff can have on the support of a sense of home. By looking at the various perspectives, a richer dataset comes to the fore, which also includes the more facilitating aspects, rather than a focus on outcomes solely.

Although the themes showed a great overlap between the groups of stakeholders, there were many subtle and more prominent differences, in which different aspects were stressed or mentioned. These supplementary views help to understand the complex phenomenon of the sense of home. For instance, in relation to the quality of care, some residents seemed reluctant to critically address or reflect on the aspects of quality of care. The current study shows that the relationship and daily interactions with staff is not always friendly or amicable in character, but rather functional and social in nature. The research team found that there is a taboo on critically discussing the subject of quality of care, as residents wish to maintain good relationships with staff as they experience a dependency on these professionals. This dependence is hardly mentioned by the residents, although it is a frequently discussed topic among relatives. Next to professional competence, quality of care is highly influenced by the care relationship. Communication, having an open dialogue, empathy and expectations all influence the relationship. Although the care professionals in our study all say to place the well-being of the resident at the center of their care, in practice this is not the reality as perceived by residents and their relatives. This highlights the need for tools to facilitate the discussion on quality of care between residents, their relatives and professional caregivers. Currently, this is predominantly focused on physical health related aspects, such as indicators for pressure ulcers, incontinence or falls, but new indicators and methodologies should be explored to facilitate the dialogue. Feeling at home may be an essential element for providing person centred care and may have an impact on contributing to quality of care [31, 32].

Having a private room was deemed important by all three groups, although it seemed that relatives valued having a private room more than the residents did. The idea of a parent having to share a room with a co-resident and the child not being able to have private conservation with a parent seemed to be a worrisome notion. Having the opportunity to decorate the private room according to personal preferences was mentioned by all three groups, although practice learns that this is not always facilitated in a liberal fashion. Van Hoof et al. [9] showed that personal belongings can help develop a sense of home among residents of Dutch nursing homes, and that residents with larger rooms have better opportunities to bring goods from their former homes. Among the building-related positive attributes to a sense of home were familiar environments, being together, being able to go outdoors, and having some degree of control over one´s own life. Negative attributes were long corridors, storage spaces, and static interior design and decoration. Another statement made by residents was that they “felt like being in a hotel”, which is very different from a sense of home. It reflects the dual nature of a nursing home as a place of residence (not home per se), and a place for receiving care services.

The current study also showed that the nursing home may be a house to the residents, it is not necessarily a home, too. In contrast to the own home people used to dwell, there are many unfamiliar faces around, there are relatives of co-residents who are there to visit them, and there are changes in staff. At home, you commonly know the people that pass through your front door. Household activities are carried out by others, for instance, cleaning staff. At home, there is a seemingly full autonomy about the things people do and don´t. To date, there are many assumptions being made in nursing homes, for instance, that residents should be stimulated to help in activities and do household tasks. This study showed that many of these assumptions may not be entirely valid for the total nursing home population, which may hamper their development of a sense of home, and, in turn, quality of life. Many of these assumptions are made by relatives and care professionals, and these need to be further verified in future studies. Despite the best of intentions, some of these assumptions and opinions may not be shared by the residents, for instance, the ´adversary´ effects of regulations, the need for measures to improve safety and security, and the need for hygiene. Such measures may limit the degree of freedom of residents, and be a cause of frictions. Among the positive things that should be maintained, according to the three groups of stakeholders, were the large selection of activities (watching TV, sports, music and trips), familiarity and homelikeness, the ´sense of family´ and hospitality, the freedom to speak your mind, being able to make meals yourself, feeling a part of a group, and having animals around. Among the negative things that should be improved were having sufficient time for the resident and personal contact, not enough time to get to know each other, labelling of items and the lack of information, the lack of leisure technologies, and locking rooms and closets.

In recent years, several new alternative settings for nursing homes have been developed, focusing on small-scale, homelike care environments. Examples include Green Houses in the USA [33, 34], small-scale homelike care environments in the Netherlands [35] and green care farms [36]. In these homes, a radical redesign of the physical, social and organizational environment has been implemented, much according to the findings of our study indicating important factors contributing to a homelike feeling. Evidence suggests that promoting a sense of self and normality, for example knowing the person, welcoming family in the nursing home, providing meaningful activities, being in a personalized environment and experiencing flexibility and continuity are perceived as highly positive in these environments [31, 37].

### Reflections on the methodology: strengths and limitations

In the current study, photographs were taken first after which interviews were conducted. Sometimes there was a substantial period of time between them (about two weeks). In future studies, the researcher could undertake the photography together with the participants and conduct the interviews directly afterwards, or even during the photography sessions. The participants will be able to remember why a certain picture was taken in the first instance and provide a better description. This is particularly true when it takes some weeks to have the photographs developed (as traditional photography is getting out of fashion and is no longer optimally supported by the market chain). Using a digital camera is more costly, but solves the challenge of having to wait for the development of photos. In addition, using disposable cameras resulted in half of the photographs being blurred and, therefore, not useful for the study. When taking photographs together with the participants, one can do a secondary check with the participants by again showing the photographs and the transcripts in a later stage as a form of member check. In the current study, individual interviews and focus group sessions were conducted. In the focus group sessions, there was less space for individuals to share their experiences and thoughts, although the mutual exchange of ideas were a source of inspiration and yielded direct take home messages for the participants. This methodology reflected the approach know as a photovoice study [20]. In addition, for future studies we propose that the method be repeated with instructions to take photos of experiences that contribute to the sense of home or detract from it; the literature suggests that experiences of “not home” or “homelessness” are powerful detractors to the global sense of home.

In this study, both residents with a psychogeriatric, physical or combined diagnosis for admission were included. No distinction was made between the levels of cognitive functioning, for instance, due to the presence of dementia syndrome by considering the outcomes of the Mini Mental State Examination [38]. The same is true for depressive symptoms, which are commonly found in a large portion of nursing home residents [39, 40]. Depressive symptoms among nursing home residents are correlated with unmet and global needs [40]. In future studies, a distinction may be made between residents with various stages of dementia and depressive symptoms, and how these symptoms may impact the sense of home, coping behaviours and adaptation, and the way the home environment is shaped. This recommendation is based on the fact that some participants from the three groups indicated that there were differences in the way wards were decorated. In psychogeriatric nursing home wards, there was more attention spent on decoration and having nostalgic items around. In a ward for physically impaired residents, there was a focus on photographs and activities and trips. Some of the care professionals indicated that by participating and taking photographs, they became more sensitized about the role of the physical environment. It made them aware of both positive and negative aspects on the work floor. Therefore, the strategy may also be used for sensitizing processes on the work floor, although the overall effectiveness and the sustainability of changes is unknown. Some relatives provided feedback that taking 25 photographs was much, and that they took random photos in the end in order to fill up the reel. Some residents indicated that they thought the phenomenon studied, i.e., the sense of home, was complex. Some people took pictures of objects, for instance a television set, but spent hardly any attention to this technology during the interview. One should question the value and meaning of such photographs for the purpose of the study.

## Conclusion

This study found that a familiar interior design and building, eating and drinking in ambience, having autonomy and control over daily life, involvement of relatives, being engaged with others and in activities and having a good quality of care are important themes, identified by all three groups of stakeholders, in experiencing a sense of home in the nursing home. Nursing staff did not focus on the role that nature, the outdoors and coping play in the establishment of a sense of home, which provides room for improvement. In addition, the themes that were identified had different meanings and connotations based on the roles the stakeholders have.
